# Comparative efficacy of adjuvant FOLFOX vs. FLOT following neoadjuvant FLOT in patients with locally advanced gastric cancer

**DOI:** 10.55730/1300-0144.6002

**Published:** 2025-06-02

**Authors:** Furkan CEYLAN, Didem ŞENER DEDE, Safa Can EFİL, Ateş Kutay TENEKECİ, Eren Göktuğ CEYLAN, Serhat SEKMEK, Mehmet ÇAKMAK, Burak BİLGİN, Şebnem YÜCEL, Hayriye TATLI DOĞAN, Mehmet Ali Nahit ŞENDUR, Muhammed Bülent AKINCI, Doğan UNCU, Bülent YALÇIN

**Affiliations:** 1 Division of Medical Oncology, Department of Internal Medicine, Faculty of Medicine, University of Health Sciences, Ankara Bilkent City Hospital, Ankara, Turkiye; 2 Division of Medical Oncology, Department of Internal Medicine, Faculty of Medicine, Yıldırım Beyazıt University, Ankara, Turkiye; 3 Faculty of Medicine, Hacettepe University, Ankara, Turkiye; 4 Division of Pulmonology, Department of Internal Medicine, Faculty of Medicine, University of Health Sciences, Ankara Bilkent City Hospital, Ankara, Turkiye; 5 Department of Pathology, Faculty of Medicine, University of Health Sciences, Ankara Bilkent City Hospital, Ankara, Turkiye

**Keywords:** Gastric cancer, FLOT, FOLFOX, response, survival

## Abstract

**Background/aim:**

Perioperative FLOT is considered the gold standard treatment for locally advanced gastric cancer. However, in the adjuvant setting, chemotherapy intolerance has brought de-escalation strategies to the forefront as an important area of research. This study aimed to compare the efficacy of adjuvant FLOT and FOLFOX regimens in enhancing survival outcomes in patients with locally advanced gastric cancer who underwent surgical resection following neoadjuvant FLOT treatment.

**Materials and methods:**

Patients with locally advanced gastric cancer (cT2-4, N0-3) who received four cycles of neoadjuvant FLOT and subsequently underwent surgery at Ankara Bilkent City Hospital between January 2018 and September 2024 were retrospectively evaluated. Disease-free survival (DFS) and overall survival (OS) outcomes were compared to patients receiving adjuvant FOLFOX or FLOT. Clinical variables such as disease stage and response to neoadjuvant therapy were also analyzed to determine their impact on DFS and OS.

**Results:**

The analysis included 171 patients, with a median age of 59 years and a median follow-up duration of 16.1 months. At 16 months, the DFS and OS rates were 66% and 82%, respectively. Of the 171 patients, 105 received adjuvant FLOT, 37 received FOLFOX, and 29 received no adjuvant therapy. Statistical analysis revealed no significant differences in DFS (HR: 0.63, 95% CI: 0.30–1.33, p = 0.229) or OS (HR: 0.76, 95% CI: 0.24–2.37, p = 0.635) between the FLOT and FOLFOX groups. However, the advanced disease stage and lack of pathological response to neoadjuvant FLOT were associated with decreased DFS and OS, highlighting these factors as potential prognostic indicators.

**Conclusion:**

Among patients undergoing surgery after neoadjuvant FLOT, adjuvant FOLFOX showed comparable efficacy to FLOT, suggesting its potential as an alternative option, particularly for patients with deteriorated ECOG PS or those who developed chemotherapy intolerance postoperatively. These findings inform treatment strategies and optimize adjuvant therapy selection based on individual patient profiles.

## 1. Introduction

Gastric cancer ranks as the fifth most prevalent cancer globally and is the third leading cause of cancer-related deaths, with a five-year survival rate of only 30%–35% [1]. Approximately 70% of cases are diagnosed at a locally advanced stage, emphasizing the aggressive progression of this disease [2]. While surgery is a cornerstone of curative treatment, evidence has consistently demonstrated that its effectiveness is significantly enhanced when combined with systemic therapies such as perioperative, adjuvant, or neoadjuvant chemotherapy or chemoradiotherapy. These approaches have been shown to improve survival outcomes, emphasizing the necessity of multimodal treatment strategies for this high-risk patient population [3–7].

However, a significant obstacle in the management of locally advanced gastric cancer is the limited tolerability of chemotherapy, particularly in the postoperative setting. This challenge is exacerbated in patients undergoing gastrectomy, where intolerance rates are notably high. Among available treatment options, the perioperative FLOT regimen (5-fluorouracil, leucovorin, oxaliplatin, and docetaxel) has emerged as the most widely accepted and Category 1 recommended approach in clinical guidelines. The FLOT4-AIO trial established its superiority in improving survival rates. Yet, it also revealed a stark reality: less than half of the patients (46%) could complete the adjuvant therapy phase due to challenges associated with its tolerability [8].

The high rates of chemotherapy intolerance have sparked growing interest in de-escalation strategies, aiming to balance treatment efficacy with patient quality of life. Unfortunately, there is a significant lack of studies focusing on adjuvant treatment options for patients who have undergone surgery following neoadjuvant therapy. This gap in evidence represents a critical unmet need, particularly as clinicians strive to optimize individualized care for this complex patient population [9–12].

Our study seeks to address this pressing issue by evaluating adjuvant treatment strategies in patients with locally advanced gastric cancer who have completed four cycles of neoadjuvant FLOT therapy followed by surgical resection. By exploring this under-researched area, we aim to contribute meaningful insights into improving treatment tolerability and outcomes, ultimately striving for a better balance between aggressive cancer control and patient well-being.

## 2. Methods

### 2.1. Patient selection

Patients diagnosed with locally advanced gastric cancer (TNM Stage: cT2-T4, N0-3) at Ankara Bilkent City Hospital between January 2018 and September 2024, who received four cycles of FLOT therapy followed by surgery, were retrospectively analyzed. Patients receiving fewer than four cycles of neoadjuvant FLOT, those treated with alternative therapies during the neoadjuvant period, and those who did not undergo surgery were excluded from the analysis.

All patients in the study received at least four cycles of the neoadjuvant FLOT regimen, which included 5-fluorouracil (2600 mg/m^2^) as a 24-h infusion on day 1, leucovorin (200 mg/m^2^) on day 1, oxaliplatin (85 mg/m^2^) on day 1, and docetaxel (50 mg/m^2^) on day 1. The regimen was administered intravenously every 2 weeks.

Participants were divided into three groups based on their adjuvant therapy. The first group consisted of patients who completed four cycles of adjuvant FLOT treatment. The second group included individuals who either did not complete FLOT or received FOLFOX therapy, while the third group comprised patients who received no adjuvant treatment.

This study was designed as a retrospective, single-center study. Medical records collected data regarding demographic and clinical characteristics, disease-free survival, and overall survival. Follow-up duration was calculated using the reverse Kaplan-Meier method. The primary endpoint was disease-free survival based on the type of adjuvant therapy received, while secondary endpoints included overall survival and factors impacting survival.

Clinical staging (cTNM) was determined using the American Joint Committee on Cancer (AJCC) 8th edition TNM staging system. To confirm staging, patients underwent computed tomography and diagnostic laparoscopy before treatment in 77% of cases.

In this study, disease-free survival was defined as the period from surgery to either relapse or death. In contrast, overall survival was measured as the time from diagnosis to death or the most recent follow-up.

### 2.2. Evaluating tumor regression

Tumor regression assessment was based on the proportion of viable tumor tissue within the macroscopically visible tumor bed or the original tumor site and was categorized into three distinct classes.

The regression grading system developed by Becker et al. [13] defines TRG1a as a complete pathological response where no viable tumor cells remain. TRG1b indicates a response with less than 10% residual tumor tissue. TRG2 reflects partial tumor regression, with 10%–50% residual tumor present. Finally, TRG3 signifies a response with more than 50% residual tumor cells, with minimal or absent signs of regression observed in the tumor bed.

### 2.3. Statistics

Descriptive statistics were reported as means with standard deviations or medians with interquartile ranges, according to the distribution of each variable. For comparisons of numerical variables, the Student’s t-test was used for normally distributed data, while the Mann-Whitney U test was applied for data without normal distribution. The Chi-Square test was employed to compare proportions among categorical variables. Disease-free and overall survival were analyzed through the Kaplan-Meier method, with group comparisons conducted using the log-rank test. Cox regression analysis identified independent predictors of survival outcomes. All statistical analyses were performed using SPSS version 26.0 (SPSS Inc., Chicago, IL, USA), and statistical significance was set at a p-value of less than 0.05.

This study received approval from the Institutional Ethics Review Board of Ankara Bilkent City Hospital and was conducted following the principles of the Declaration of Helsinki.

## 3. Results

### 3.1 Patients and tumor characteristics

The study included a total of 171 patients, with a median age of 59 years, of whom 77% were male. At diagnosis, Eastern Cooperative Oncology Group (ECOG) performance status was 0 in 40% of patients, 1 in 58%, and 2 in 2%. The tumor was located in the stomach in 66% of cases and in the gastroesophageal junction in 34%. Adenocarcinoma was the predominant histology (81%), with 64% of tumors being moderately or poorly differentiated. Tumors were staged as T3 in 69% and T4 in 25% of patients, and lymph node involvement was detected in 81%. Stage 2 disease was present in 20% of patients, stage 3 in 74%, and stage 4 in 6%. Regarding molecular characteristics, 54% of tumors were microsatellite stable, 12% had microsatellite instability. The Charlson Comorbidity Index (CCI) was below 4 in 44% of patients and 4 or higher in 56%. The most frequent presenting symptoms were dyspepsia (41%) and abdominal pain (36%), with a family history of cancer documented in 30% of cases.

Diagnostic laparoscopy was performed in 132 patients (77%), with total gastrectomy conducted in 121 patients (71%) and subtotal gastrectomy in 50 patients (29%). D2 lymphadenectomy was completed in 92% of cases, achieving an R0 resection rate of 95%. Hyperthermic intraperitoneal chemotherapy (HIPEC) was applied to 9% of patients. Tumor regression grading showed TRG1a in 8%, TRG1b in 13%, TRG2 in 19%, and TRG3 in 36% of cases. Recurrence occurred in 27% of patients, and 18% died during follow-up.

Of the 171 patients included in this study, 105 successfully completed four cycles of adjuvant FLOT therapy, while 37 received FOLFOX as an alternative due to intolerance. The decision to administer FOLFOX was based on several factors, including poor performance status (ECOG PS ≥ 3, n = 22/37), chemotherapy intolerance (n = 7/37), grade ≥3 oral intake impairment (n = 6/37), severe hematological toxicity (grade ≥ 3, n = 2/37), neuropathy (grade ≥3, n = 1/37), and acute cardiovascular events (n = 2/37). Additionally, 29 patients did not receive any adjuvant therapy, with reasons including poor ECOG PS (n = 5/29), infections (n = 5/29), patient preference (n = 5/29), surgical complications (n = 4/29), the need for adjuvant chemoradiotherapy following R1 resection (n = 3/29), and postoperative detection of distant metastasis (n = 1/29) (Table 1.).

When comparing the FLOT and FOLFOX groups, the median age at diagnosis was notably lower in the FLOT group (58 vs. 62 years, p = 0.011), and the FLOT group exhibited better ECOG performance status (ECOG 0: 50% vs. 28%; ECOG 1: 49% vs. 72%; ECOG 2: 1% vs. 0%, p = 0.003). However, patient demographics, tumor characteristics, and surgical approaches were otherwise comparable between the two groups. Comprehensive details on these parameters are provided in Table 1.

### 3.2 Survival

**Figure 1:**
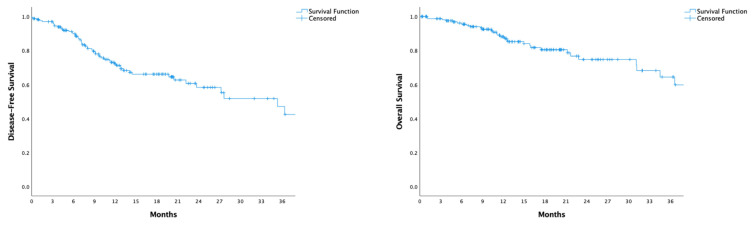
Disease-Free Survival (DFS) and Overall Survival (OS) curves for the entire cohort of patients with locally advanced gastric cancer who underwent surgery and adjuvant chemotherapy following neoadjuvant FLOT therapy. The left panel shows the DFS curve, while the right panel displays the OS curve, both measured in months. The blue line represents the survival function, and ‘+’ symbols indicate censored data points for patients who were lost to follow-up or did not experience the event by the end of the study period.

The median follow-up period was 16.1 months. Neither median disease-free survival nor overall survival was reached within this timeframe. At 16 months, disease-free survival was 66%, and overall survival was 82% (Figure 1).

**Figure 2:**
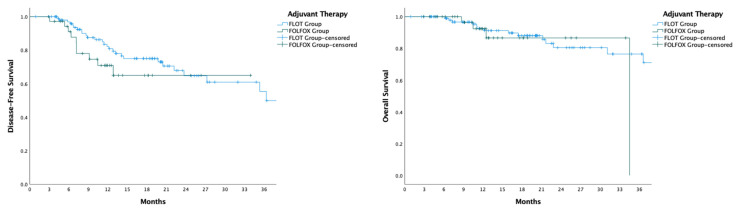
Kaplan-Meier curves comparing Disease-Free Survival (DFS) and Overall Survival (OS) between patients receiving adjuvant FLOT and FOLFOX therapy after surgery for locally advanced gastric cancer. The left panel displays the DFS curve, while the right panel shows the OS curve, both measured in months. The FLOT group is represented by the blue line, and the FOLFOX group by the green line. Censored data points, where patients were lost to follow-up or did not experience the event, are indicated by ‘+’ symbols. These curves illustrate the survival differences between the two adjuvant therapy groups over the study period.

**Figure 3:**
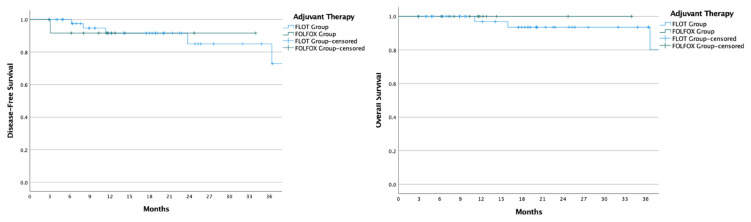
Disease-Free Survival (DFS) and Overall Survival (OS) curves for patients with tumor regression grades (TRG) 1a, 1b, and 2, comparing adjuvant FLOT and FOLFOX therapy. The left panel displays the DFS curve, while the right panel shows the OS curve, both measured in months. The FLOT group is represented by the blue line, and the FOLFOX group by the green line. ‘+’ symbols indicate censored data points where patients were lost to follow-up or did not experience the event by the end of the study. These curves illustrate survival differences between FLOT and FOLFOX in patients achieving partial or complete tumor regression.

**Figure 4:**
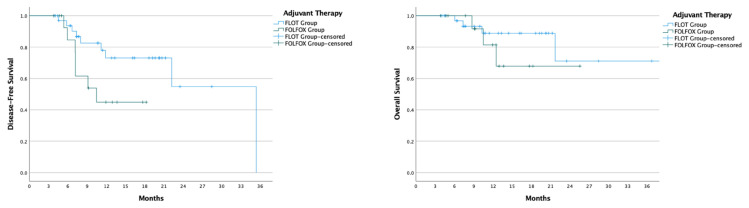
Disease-Free Survival (DFS) and Overall Survival (OS) curves for patients with tumor regression grade (TRG) 3, comparing adjuvant FLOT and FOLFOX therapy. The left panel displays the DFS curve, while the right panel shows the OS curve, both measured in months. The FLOT group is represented by the blue line, and the FOLFOX group by the green line. ‘+’ symbols indicate censored data points for patients who were lost to follow-up or did not experience the event by the end of the study. These curves illustrate the survival outcomes for TRG 3 patients, highlighting differences in DFS and OS between FLOT and FOLFOX treatment groups.

Comparison of disease-free survival between patients receiving FLOT and those receiving FOLFOX revealed similar outcomes (HR: 0.63, 95% CI: 0.30–1.33, p = 0.229), as did overall survival (HR: 0.76, 95% CI: 0.24–2.37, p = 0.635) (Figure 2). In patients who achieved a pathological response (TRG 1a, 1b, and 2), both disease-free and overall survival remained comparable between the FLOT and FOLFOX groups (DFS p = 0.971; OS p = 0.722) (Figure 3). For patients who did not achieve a pathological response (TRG 3), DFS (35.3 vs. 10.5 months, p = 0.056) and OS (NE vs. NE, p = 0.404) showed no significant differences between those receiving adjuvant FLOT and FOLFOX (Figure 4).

In the group that did not receive adjuvant chemotherapy, disease-free survival was significantly shorter than in the FLOT group (HR: 10.00, 95% CI: 4.35–25.00, p < 0.001) and the FOLFOX group (HR: 5.00, 95% CI: 1.75–14.29, p = 0.003). Overall survival was also shorter compared to both the FLOT group (HR: 9.09, 95% CI: 3.85–20.00, p < 0.001) and the FOLFOX group (HR: 6.67, 95% CI: 2.13–20.00, p = 0.001).

Factors associated with shorter disease-free survival included advanced-stage disease (Stage 4 vs. Stage 2; HR: 5.31, 95% CI: 1.70–16.53, p = 0.004), R1 resection (compared to R0 resection; HR: 3.91, 95% CI: 1.53–9.98, p = 0.004), and failure to achieve a pathological response (TRG 3 vs. TRG 1a, 1b, 2; HR: 5.56, 95% CI: 2.38–14.29, p < 0.001). Shorter overall survival was similarly associated with advanced-stage disease (Stage 4 vs. Stage 2; HR: 7.33, 95% CI: 1.73–31.02, p = 0.007), R1 resection (vs. R0 resection; HR: 5.35, 95% CI: 1.56–18.27, p = 0.008), poorly cohesive carcinoma (HR: 2.63, 95% CI: 1.28–5.56, p = 0.009), and failure to achieve a pathological response (HR: 7.69, 95% CI: 2.33–25.00, p < 0.001) (Tables 2 and 3).

In multivariate analysis for disease-free survival, advanced-stage disease (Stage 4 vs. Stage 2; HR: 8.52, 95% CI: 1.70–42.77, p < 0.001) and failure to achieve a pathological response (TRG 3 vs. TRG 1a, 1b, and 2. HR: 5.56, 95% CI: 2.33–14.29, p < 0.001) were significant predictors of shorter survival. Similarly, in multivariate analysis for overall survival, advanced-stage disease (Stage 4 vs. Stage 2; HR: 5.65, 95% CI: 2.32–13.74, p = 0.009) and failure to achieve a pathological response (TRG 3 vs. TRG 1a, 1b, and 2. HR: 7.69, 95% CI: 2.17–25.00, p = 0.002) remained significant factors associated with shorter survival (Table 2, 3).

## 4. Discussion

This study revealed that adjuvant FLOT and FOLFOX regimens yielded comparable outcomes in terms of DFS and OS in patients with locally advanced gastric cancer who underwent surgical resection following four cycles of neoadjuvant FLOT therapy. Notably, advanced disease stage and an inadequate pathological response emerged as significant predictors of reduced DFS and OS. Furthermore, patients who were unable to receive adjuvant chemotherapy experienced markedly shorter survival.

For resectable locally advanced gastric cancers, perioperative chemotherapy remains the cornerstone of treatment, offering the most favorable survival outcomes [8, 14]. Among eligible patients with good performance status, minimal comorbidities, and the ability to tolerate intensive regimens, the FLOT regimen has demonstrated superiority over ECF/ECX, with a median survival advantage of 50 months versus 35 months (p = 0.012), as evidenced in the FLOT4/AIO trial [8]. However, the trial also highlighted the regimen’s toxicity, with frequent grade 3 or higher adverse events, including infections, diarrhea, peripheral neuropathy, and nausea. These toxicities, along with disease progression and chemotherapy intolerance, resulted in only 46% of patients completing all four adjuvant FLOT cycles, showing the pressing need for deescalation strategies. Interestingly, our study demonstrated a significantly higher completion rate (64%) for the FLOT regimen, exceeding the completion rates reported in both the FLOT4 and MAGIC trials [5, 8]. These findings highlight the potential to refine treatment protocols further, balancing efficacy with patient quality of life, a critical consideration in gastric cancer management.

Identifying patients who are unlikely to derive significant benefit from intensive adjuvant chemotherapy is a critical step toward optimizing treatment strategies, as it enables the targeted application of deescalation approaches. This strategy not only minimizes unnecessary chemotherapy-related toxicities but also aligns with the principles of personalized medicine by tailoring treatment decisions to individual patient profiles. In the context of gastric cancer, the relevance of de-escalation strategies is particularly pronounced during the adjuvant phase, where treatment intolerance is a common challenge. Ongoing research aims to pinpoint subsets of patients who may not benefit from adjuvant chemotherapy after neoadjuvant treatment and surgical resection, addressing a significant gap in the current literature. One promising avenue is the study of circulating tumor DNA (ctDNA), which has already demonstrated its utility in predicting the need for and efficacy of adjuvant therapies in breast and lung cancer [15–17]. Efforts are underway to evaluate its prognostic and predictive potential in gastric cancer. Moreover, de-escalation strategies informed by pathological response and lymph node ratio (LNR) are gaining increasing recognition as practical and evidence-based approaches [18, 19]. These advancements hold the potential to refine adjuvant treatment paradigms, striking a balance between maximizing survival outcomes and minimizing undue patient burden.

The study by Lin et al. demonstrated that adjuvant chemotherapy significantly improved survival outcomes in patients with a metastatic lymph node ratio exceeding 9%, whereas no survival benefit was observed in those with a ratio below this threshold [19]. These findings highlight the critical role of thorough lymph node dissection and the presence of lymph node metastases in determining the effectiveness of adjuvant therapy. Additionally, the results suggest that tumor shrinkage could serve as a valuable prognostic and predictive marker, aiding in treatment decisions and identifying patients most likely to benefit from adjuvant interventions.

Several retrospective studies have investigated the impact of adjuvant therapy on survival outcomes based on pathological response rates. Liu et al. [18] reported that adjuvant FLOT does not confer additional survival benefits for patients with either a complete or absent pathological response, supporting a more tailored approach to treatment. In patients with poor pathological response following neoadjuvant therapy, DFS and OS were comparable between those receiving adjuvant FLOT and those who did not (DFS HR: 1.03, 95% CI: 0.78–1.36; OS HR: 0.96, 95% CI: 0.70–1.30). Similarly, for patients achieving a complete pathological response, survival outcomes remained consistent regardless of adjuvant FLOT administration (DFS HR: 0.88, 95% CI: 0.41–1.85; OS HR: 0.69, 95% CI: 0.31–1.54). However, among patients with partial pathological response, those treated with adjuvant FLOT exhibited significantly improved DFS and OS (DFS HR: 0.68, 95% CI: 0.55–0.86; OS HR: 0.55, 95% CI: 0.44–0.69) compared to their untreated counterparts. In contrast, the study by Lin et al. in China found no survival benefit associated with adjuvant therapy in patients who achieved a pathological response [10]. Conversely, Mokdad et al. reported favorable outcomes with adjuvant therapy even in patients classified as ypT0 (HR: 0.63) or ypN0 (HR: 0.68) [12]. These discrepancies highlight the complexity of tailoring adjuvant therapy based on pathological response and shows the need for further research to refine treatment strategies.

In our analysis, patients with or without a pathological response exhibited comparable disease-free and overall survival outcomes between the FLOT and FOLFOX groups. This observation aligns with the findings of Liu et al., where both complete and absent pathological responses were associated with similar survival outcomes, irrespective of the adjuvant regimen used [18]. However, unlike Liu et al. [18], our study did not identify a survival advantage for patients with intermediate pathological responses receiving FLOT. These findings are consistent with two other large retrospective studies in the literature, further emphasizing the variability in survival outcomes across different pathological response groups [10, 12]. Prior studies have highlighted the prognostic significance of tumor regression as an indicator of survival outcomes [20, 21]. Liu et al.’s work shows the importance of tailoring treatment decisions based on response, contributing valuable insights into personalized therapy strategies [18]. In our study, while tumor regression served as a prognostic marker for both disease-free and overall survival, it did not appear to influence the choice of adjuvant therapy. Patients who did not receive adjuvant chemotherapy experienced significantly shorter survival compared to those who underwent treatment, largely due to factors such as surgical complications, infections, and early mortality, which impeded access to adjuvant therapy. While this highlights the critical role of adjuvant treatment in improving outcomes, our study primarily centers on exploring de-escalation strategies rather than evaluating the decision to administer adjuvant therapy. These findings underline the need for prospective studies to assess the efficacy of adjuvant therapy tailored to pathological response, which could pave the way for more individualized treatment approaches in gastric cancer. The CLASSIC trial, a pivotal study supporting the use of adjuvant fluoropyrimidine and oxaliplatin after D2 gastrectomy, demonstrated significant improvements in both DFS and OS compared to observation (5-year OS: 68% vs. 53%). However, high toxicity rates were reported, with grade ≥3 adverse events in 56% of patients, dose reductions in 90%, and treatment discontinuation in 10%, primarily due to neutropenia. These findings underline both the efficacy and the tolerability challenges of oxaliplatin-based adjuvant therapy [22]. In our study, FOLFOX was administered to patients with limited postoperative tolerance, yet yielded survival outcomes comparable to FLOT. This suggests that maintaining treatment feasibility, even with a less intensive regimen, may be more critical than maximal dose intensity in certain real-world scenarios. In a phase II study by Wang et al., the efficacy and safety of perioperative FOLFOX were evaluated in 73 patients with locally advanced gastric cancer. The study reported a 3-year overall survival rate of 62%, with a relatively low incidence of grade ≥3 adverse events (13.7%). These results suggest that FOLFOX may offer a favourable balance between efficacy and tolerability in the perioperative setting [23]. Consistent with these findings, our study demonstrated that adjuvant FOLFOX provided comparable survival outcomes to FLOT in a postoperative population with poorer clinical status, further supporting its role as a less toxic yet effective alternative in selected patients. Li et al. compared perioperative FOLFOX with adjuvant-only FOLFOX in patients with locally advanced gastric cancer. Among 73 patients, the perioperative approach achieved a higher overall response rate (70%) and significantly improved 4-year overall survival (78% vs. 51%, p = 0.031). Notably, grade ≥3 adverse events were observed in only 4% of patients. These findings support the survival benefit and safety of oxaliplatin-based perioperative regimens [24]. In line with these results, our study suggests that even when used solely in the adjuvant setting, FOLFOX may provide meaningful survival outcomes in patients who are unable to tolerate triplet therapy, further underscoring its value as a feasible alternative. Chen et al. compared neoadjuvant EOX and FOLFOX in patients with locally advanced gastric cancer and found that EOX yielded a higher objective response rate but was associated with a significantly higher incidence of grade ≥3 adverse events (30% vs. 10%) [25]. In contrast, our study focused on the adjuvant setting and demonstrated that FOLFOX maintained favourable survival outcomes with an acceptable safety profile, supporting its use in patients with limited tolerance for more intensive regimens such as FLOT or EOX.

These findings highlight the trade-off between efficacy and tolerability when selecting preoperative regimens. Ultimately, while triplet-based regimens may offer superior objective response rates and long-term survival, their completion remains challenging for many patients, particularly in the adjuvant setting, where postoperative vulnerability often necessitates a more individualized and tolerability-driven therapeutic approach.

Taken together, the heterogeneity of findings across studies underscores the multifactorial nature of adjuvant chemotherapy benefit in gastric cancer. While some data support tailoring treatment based on pathological response or lymph node involvement, others suggest that adjuvant therapy may confer benefit even in complete responders. These discrepancies likely reflect differences in study design, patient selection, surgical quality (e.g., extent of lymphadenectomy), and the timing, tolerability, and intensity of systemic therapy. Our study, based on a real-world cohort, contributes to this discussion by demonstrating that survival outcomes were comparable between patients receiving adjuvant FLOT and those receiving FOLFOX, despite the latter group including older individuals with worse ECOG performance status and higher comorbidity burden.

Notably, our findings showed no significant difference in disease-free or overall survival even among patients with partial pathological response (TRG 2), which challenges previous suggestions that such patients may preferentially benefit from more intensive adjuvant regimens. In our cohort, decisions to administer FOLFOX were driven primarily by clinical deterioration after surgery, such as ECOG PS ≥ 2, oral intake difficulties, or hematological toxicity, rather than by pathological staging alone. This reflects real-world treatment dynamics, where regimen tolerability often supersedes idealised treatment planning. The fact that FOLFOX yielded outcomes comparable to FLOT in this context suggests that the capacity to deliver any postoperative chemotherapy, even if less intensive, may be more important than adhering rigidly to triplet regimens.

Moreover, among patients who did not achieve a pathological response (TRG 3), survival remained similarly poor regardless of whether FLOT or FOLFOX was used, further indicating that chemotherapy escalation may not compensate for underlying tumour resistance. This raises the question of whether more nuanced approaches, such as biomarker-guided treatment, ctDNA monitoring, or immunologic profiling, could better identify which patients truly benefit from adjuvant therapy. Future prospective trials that stratify patients not only by pathological features but also by dynamic indicators of minimal residual disease are warranted to refine adjuvant strategies and prevent overtreatment in subgroups unlikely to benefit.

The retrospective design of our study stands as a key limitation, as it inherently restricts our ability to control confounding variables and introduces potential selection bias. Moreover, although chemotherapy intolerance was the principal driver of adjuvant therapy de-escalation, adverse events were not systematically captured across the cohort. While validated quality of life assessments was unavailable, we utilized clinical proxies such as ECOG performance status deterioration and treatment-related hospitalizations to reflect functional decline. Notably, several patients in the FOLFOX group required inpatient care due to fatigue, gastrointestinal complications, neutropenic fever, or cardiovascular events (Table 4). In addition to these limitations, the relatively short median follow-up period (16.1 months) and the smaller sample sizes in the FOLFOX and no-adjuvant groups may reduce our findings’ statistical power and generalizability. Nevertheless, despite these constraints, our study provides meaningful real-world insights into the feasibility and outcomes of adjuvant therapy deescalation in gastric cancer. These findings underscore the importance of developing more personalized and tolerable treatment strategies, and we believe they offer a valuable foundation for future prospective research with longer follow-up and more robust quality of life assessment tools.

## Conclusion

In conclusion, our study demonstrates that adjuvant FOLFOX is a practical alternative to FLOT for patients with locally advanced gastric cancer who undergo surgery after neoadjuvant FLOT, particularly for those with reduced performance status or intolerance to intensive postoperative chemotherapy. The use of FOLFOX in such cases preserves survival benefits while potentially minimizing treatment-related adverse effects.

This flexibility highlights the importance of individualized treatment strategies in the adjuvant setting, allowing effective oncologic care to be tailored to patients’ specific needs and tolerances. By focusing on personalized approaches, this strategy aims to optimize clinical outcomes while enhancing the quality of life during the challenging postoperative period, marking progress toward more patient-centered gastric cancer care.

## Figures and Tables

**Table 1:** Baseline clinical and tumor characteristics of the patient cohort.

		Whole (n = 171)	FLOT group (n = 105)	FOLFOX group (n = 37)	No Chemo (n = 29)	p-value
Age, Median, IQR		59 (51–67)	58 (49–64)	62 (55–69)	63 (50–68)	0.011
Sex, Male		132 (77%)	79 (75%)	28 (78%)	25 (86%)	0.412
ECOG	0	69 (40%)	53 (50%)	10 (28%)	6 (21%)	0.003
	1	99 (58%)	52 (49%)	26 (72%)	21 (72%)	
	2	3 (2%)	1 (1%)	0 (0%)	2 (7%)	
Localization	GEJ	58 (34%)	35 (33%)	13 (36%)	10 (35%)	0.942
	Gastric	113 (66%)	71 (67%)	23 (64%)	19 (65%)	
Histology	Adenocarcinoma	139 (81%)	87 (82%)	30 (83%)	22 (76%)	0.703
	Poorly cohesive	32 (19%)	19 (18%)	6 (17%)	7 (24%)	
Grading according to the WHO	Unknown	46 (27%)	30 (28%)	10 (28%)	6 (21%)	0.468
	Grade 1	16 (9%)	9 (8%)	6 (17%)	1 (3%)	
	Grade 2	41 (24%)	23 (22%)	9 (25%)	9 (31%)	
	Grade 3	68 (40%)	44 (42%)	11 (30%)	6 (21%)	
Stage T	T1	0 (0%)	0 (0%)	0 (0%)	0 (0%)	0.857
	T2	11 (6%)	7 (7%)	2 (6%)	2 (7%)	
	T3	118 (69%)	73 (69%)	27 (75%)	18 (62%)	
	T4	42 (25%)	26 (24%)	7 (19%)	9 (31%)	
Stage N	N0	33 (19%)	22 (21%)	8 (22%)	3 (10%)	0.726
	N1	81 (47%)	50 (47%)	14 (39%)	17 (59%)	
	N2	44 (26%)	27 (25%)	10 (28%)	7 (24%)	
	N3	13 (7%)	7 (7%)	4 (11%)	2 (7%)	
Stage	2	35 (20%)	23 (22%)	8 (22%)	4 (14%)	0.730
	3	126 (74%)	78 (74%)	26 (72%)	22 (76%)	
	4	10 (6%)	5 (5%)	2 (6%)	3 (10%)	
Microsatellite Status	Unknown	59 (34%)	42 (40%)	9 (25%)	8 (27%)	0.085
	MSS	92 (54%)	47 (44%)	26 (70%)	19 (66%)	
	MSI-H	21 (12%)	17 (8%)	2 (5%)	2 (7%)	
Charlson Comorbidity Index	<4	75 (44%)	51 (48%)	13 (36%)	11 (38%)	0.355
	≥4	96 (56%)	55 (52%)	23 (64%)	18 (62%)	
Family history		51 (30%)	31 (29%)	10 (28%)	10 (35%)	0.823
Smoking		84 (49%)	43 (41%)	25 (70%)	16 (55%)	0.009
Alcohol		9 (6%)	3 (3%)	4 (11%)	2 (7%)	0.144
Chief complaint	Dyspepsia	68 (41%)	44 (43%)	16 (44%)	8 (28%)	0.294
	Abdominal pain	61 (36%)	37 (36%)	12 (33%)	12 (41%)	0.792
	Weight loss	8 (5%)	4 (4%)	2 (6%)	2 (7%)	0.750
	Hematemesis/ Melena	14 (8%)	8 (8%)	3 (8%)	3 (10%)	0.906
	Dysphagia	27 (16%)	16 (15%)	5 (14%)	6 (21%)	0.719
Diagnostic laparoscopy		132 (77%)	80 (76%)	30 (83%)	22 (76%)	0.613
Surgery	Total gastrectomy	121 (71%)	77 (73%)	23 (64%)	21 (72%)	0.594
	Subtotal gastrectomy	50 (29%)	29 (27%)	13 (36%)	8 (28%)	
Dissection	D1	14 (8%)	8 (7%)	2 (6%)	4 (14%)	0.449
	D2	157 (92%)	98 (93%)	34 (94%)	25 (86%)	
Resection	R0	163 (95%)	103 (97%)	33 (92%)	27 (93%)	0.331
	R1	8 (5%)	3 (3%)	3 (8%)	2 (7%)	
Use of HIPEC		15 (9%)	6 (6%)	5 (14%)	4 (14%)	0.185
Number of lymph nodes excised, Median, IQR		26 (21–35)	27 (21–36)	25 (21–34)	24 (17–34)	0.758
ypT	ypT0	17 (10%)	11 (10%)	4 (11%)	2 (7%)	0.152
	ypT1	24 (14%)	16 (15%)	7 (20%)	1 (3%)	
	ypT2	17 (10%)	14 (13%)	0 (0%)	3 (10%)	
	ypT3	85 (50%)	49 (46%)	21 (58%)	15 (52%)	
	ypT4	28 (16%)	16 (15%)	4 (11%)	8 (28%)	
ypN	ypN0	83 (49%)	57 (54%)	15 (42%)	11 (38%)	0.273
	ypN1	32 (19%)	20 (19%)	7 (19%)	5 (17%)	
	ypN2	28 (16%)	16 (15%)	8 (22%)	4 (14%)	
	ypN3	28 (16%)	13 (12%)	6 (17%)	9 (31%)	
Metastases	ypM1	8 (5%)	4 (4%)	1 (3%)	3 (10%)	0.276
Tumor Regression Score	TRG X	41 (24%)	24 (23%)	8 (22%)	9 (31%)	0.646
	TRG 1a	14 (8%)	10 (9%)	2 (6%)	2 (7%)	
	TRG 1b	22 (13%)	13 (12%)	6 (17%)	3 (10%)	
	TRG 2	32 (19%)	24 (23%)	4 (11%)	4 (14%)	
	TRG 3	62 (36%)	35 (33%)	16 (44%)	11 (38%)	
Relapse		46 (27%)	25 (24%)	8(22%)	13 (45%)	0.057
Death		31 (18%)	15 (14%)	4 (11%)	12 (41%)	0.002

Table 1 presents the baseline clinical and tumor characteristics of the patient cohort, including demographic data, tumor location, histology, tumor grading, TNM staging according to the American Joint Committee on Cancer (AJCC) guidelines, microsatellite status (microsatellite stable [MSS], microsatellite instability-low [MSI-L], or microsatellite instability-high [MSI-H]), Charlson Comorbidity Index (CCI), family cancer history, smoking and alcohol use, primary symptoms, surgical approach, extent of lymph node dissection (D1 or D2), resection margin status (R0 or R1), administration of hyperthermic intraperitoneal chemotherapy (HIPEC), lymph node excision count, and tumor regression grade (TRG). Patients were grouped by adjuvant therapy type, including those who completed adjuvant FLOT (5-fluorouracil, leucovorin, oxaliplatin, and docetaxel), those who received FOLFOX (5-fluorouracil, leucovorin, and oxaliplatin) due to intolerance, and those who received no adjuvant therapy. Disease-free survival (DFS) and overall survival (OS) outcomes were measured, with significance values provided to illustrate differences across groups. The Eastern Cooperative Oncology Group Performance Status (ECOG PS) was also recorded, alongside interquartile range (IQR) measures where appropriate.

**Table 2:** Univariate and multivariate analysis for disease-free survival.

	Univariate			Multivariate		
	HR	95% CI	p-values	HR	95% CI	p-values
Age (<65 vs. ≥65)	0.87	0.49–1.54	0.642			
Male/female	0.94	0.48–1.83	0.844			
**ECOG 0 vs. 1&2**	**0.64**	**0.36**–**1.14**	**0.131**			
CCI (<4 vs. ≥4)	0.88	0.51–1.51	0.638			
**Adenocarcinoma vs. poorly cohesive**	**0.64**	**0.34**–**1.18**	**0.149**			
Grade 1 & 2 vs. 3	0.99	0.54–1.83	0.976			
MSS vs. MSI-H	1.19	0.51–2.75	0.693			
D2 vs. D1 dissection	0.62	0.26–1.45	0.270			
**R1 vs R0 resection**	**3.91**	**1.53**–**9.98**	**0.004**			
**Stage 3 vs. 2**	**2.30**	**0.97**–**5.44**	**0.057**	2.57	0.59–11.23	0.208
**Stage 4 vs. 2**	**5.31**	**1.70**–**16.53**	**0.004**	**8.52**	**1.70**–**42.77**	**<0.001**
Localization (GEJ vs. gastric)	1.35	0.78–2.35	0.287			
**TRG 1a-1b-2 vs. 3**	**0.18**	**0.07**–**0.42**	**<0.001**	**0.18**	**0.07**–**0.43**	**<0.001**
**Adjuvant Therapy**						
FLOT vs. FOLFOX	0.63	0.30–1.33	0.229			

Table 2 shows the results of univariate and multivariate analyses for disease-free survival (DFS) in the patient cohort, including hazard ratios (HR) with 95% confidence intervals (CI) and p-values for each variable. Variables assessed include age (<65 vs. ≥65), gender (male vs. female), Eastern Cooperative Oncology Group Performance Status (ECOG; 0 vs. 1&2), Charlson Comorbidity Index (CCI; <4 vs. ≥4), histology (adenocarcinoma vs. poorly cohesive carcinoma), tumor grade (Grade 1&2 vs. Grade 3), microsatellite status (microsatellite stable [MSS] vs. microsatellite instabl [MSI-H]), extent of lymph node dissection (D1 vs. D2), resection margin status (R0 vs. R1), disease stage (Stage 3 vs. 2, Stage 4 vs. 2), tumor localization (gastroesophageal junction [GEJ] vs. gastric), tumor regression grade (TRG; 1a–1b–2 vs. 3), and type of adjuvant therapy (FLOT vs. FOLFOX). These factors were evaluated to identify potential predictors of DFS, with multivariate analysis adjusting for confounding variables to determine independent predictors of survival outcomes.

**Table 3:** Univariate and multivariate analysis for overall survival.

	Univariate			Multivariate		
	HR	95% CI	p-values	HR	95% CI	p-values
Age (<65 vs. ≥65)	1.02	0.48–2.15	0.967			
**Male/female**	**0.50**	**0.24**–**1.06**	**0.072**			
ECOG 0 vs. 1&2	0.69	0.34–1.43	0.319			
CCI (<4 vs. ≥4)	1.00	0.50–2.00	0.992			
**Adenocarcinoma vs. poorly cohesive**	**0.38**	**0.18**–**0.78**	**0.009**			
Grade 1 & 2 vs. 3	0.75	0.34–1.65	0.477			
MSS vs. MSI-H	1.21	0.46–3.19	0.695			
**D2 vs. D1 dissection**	**0.42**	**0.16**–**1.09**	**0.075**			
**R1 vs. R0 resection**	**5.35**	**1.56**–**18.27**	**0.008**			
**Stage 3 vs. 2**	**2.35**	**0.71**–**7.81**	**0.164**	2.57	0.59–11.23	0.208
**Stage 4 vs. 2**	**7.33**	**1.73**–**31.02**	**0.007**	**5.65**	**2.32**–**13.74**	**0.009**
Localization (GEJ vs. gastric)	0.92	0.44–1.95	0.831			
**TRG 1a**–**1b**–**2 vs. 3**	**0.13**	**0.04**–**0.43**	**<0.001**	**0.13**	**0.04**–**0.46**	**0.002**
**Adjuvant Therapy**						
**FLOT vs. FOLFOX**	**0.76**	**0.24**–**2.37**	**0.635**			

Table 3 shows the univariate and multivariate analyses for overall survival (OS) in the patient cohort, with hazard ratios (HR), 95% confidence intervals (CI), and p-values reported for each variable. Variables examined include age (<65 vs. ≥65), gender (male vs. female), Eastern Cooperative Oncology Group Performance Status (ECOG; 0 vs. 1&2), Charlson Comorbidity Index (CCI; <4 vs. ≥4), histology (adenocarcinoma vs. poorly cohesive carcinoma), tumor grade (Grade 1&2 vs. Grade 3), microsatellite status (microsatellite stable [MSS] vs. microsatellite instable [MSI-H]), extent of lymph node dissection (D1 vs. D2), resection margin status (R0 vs. R1), disease stage (Stage 3 vs. 2 and Stage 4 vs. 2), tumor localization (gastroesophageal junction [GEJ] vs. gastric), tumor regression grade (TRG; 1a-1b–2 vs. 3), and adjuvant therapy type (FLOT vs. FOLFOX). This table highlights both unadjusted and adjusted analyses to determine independent predictors of OS, focusing on variables significantly associated with survival outcomes.

**Table 4:** Adverse events according to the group.

FOLFOX Group (n = 37)		No Chemo (n = 29)	

Chemotherapy intolerance	7	Infection	5
Fatigue	7		
Diarrhea	3		
Neuropathy	1		

ECOG PS ≥ 3	22	Surgical complication	4

Oral intake impairment	6	ECOG PS ≥ 3	5

Neutropenic fever	2	Patient preference	5

Acute cardiovascular events	2	Received adjuvant chemoradiotherapy	3

Grade ≥ 3 hematological toxicity	2	Postoperative detection of distant metastasis	1

		Early exitus	4

Table 4 summarizes the clinical factors that led to the initiation of FOLFOX (n = 37) or to withholding adjuvant chemotherapy entirely (n = 29). ECOG PS: Eastern Cooperative Oncology Group Performance Status.

## References

[1] Smyth EC Nilsson M Grabsch HI van Grieken NC Lordick F Gastric cancer Lancet 2020 396 10251 635 648 10.1016/S0140-6736(20)31288-5 32861308

[2] Zeng H Chen W Zheng R Zhang S Ji JS Changing cancer survival in China during 2003–15: a pooled analysis of 17 population-based cancer registries Lancet Global Health 2018 6 5 e555 e567 10.1016/S2214-109X(18)30127-X 29653628

[3] Macdonald John S Smalley Stephen R Benedetti J Hundahl Scott A Estes Norman C Chemoradiotherapy after Surgery Compared with Surgery Alone for Adenocarcinoma of the Stomach or Gastroesophageal Junction New England Journal of Medicine 345 10 725 730 10.1056/NEJMoa010187 11547741

[4] Alderson D Cunningham D Nankivell M Blazeby JM Griffin SM Neoadjuvant cisplatin and fluorouracil versus epirubicin, cisplatin, and capecitabine followed by resection in patients with oesophageal adenocarcinoma (UK MRC OE05): an open-label, randomised phase 3 trial Lancet Oncology 2017 18 9 1249 1260 10.1016/S1470-2045(17)30447-3 28784312 PMC5585417

[5] Cunningham D Allum William H Stenning Sally P Thompson Jeremy N Van de Velde Cornelis JH Perioperative Chemotherapy versus Surgery Alone for Resectable Gastroesophageal Cancer New England Journal of Medicine 355 1 11 20 10.1056/NEJMoa055531 16822992

[6] Ychou M Boige V Pignon JP Conroy T Bouché O Perioperative chemotherapy compared with surgery alone for resectable gastroesophageal adenocarcinoma: an FNCLCC and FFCD multicenter phase III trial Journal of Clinical Oncology 2011 29 13 1715 1721 10.1200/JCO.2010.33.0597 21444866

[7] van Hagen P Hulshof MCCM van Lanschot JJB Steyerberg EW Henegouwen MIvB Preoperative Chemoradiotherapy for Esophageal or Junctional Cancer New England Journal of Medicine 366 22 2074 2084 10.1056/NEJMoa1112088 22646630

[8] Al-Batran SE Homann N Pauligk C Goetze TO Meiler J Perioperative chemotherapy with fluorouracil plus leucovorin, oxaliplatin, and docetaxel versus fluorouracil or capecitabine plus cisplatin and epirubicin for locally advanced, resectable gastric or gastro-oesophageal junction adenocarcinoma (FLOT4): a randomised, phase 2/3 trial Lancet 2019 393 10184 1948 1957 10.1016/S0140-6736(18)32557-1 30982686

[9] Tong X Zhi P Lin S Neoadjuvant Chemotherapy in Asian Patients With Locally Advanced Gastric Cancer Journal of Gastric Cancer 2023 23 1 182 193 10.5230/jgc.2023.23.e12 36750998 PMC9911622

[10] Lin JX Tang YH Lin GJ Ma YB Desiderio J Association of Adjuvant Chemotherapy With Overall Survival Among Patients With Locally Advanced Gastric Cancer After Neoadjuvant Chemotherapy JAMA Network Open 2022 5 4 e225557 10.1001/jamanetworkopen.2022.5557 35363268 PMC8976237

[11] Cho H Nakamura J Asaumi Y Yabusaki H Sakon M Long-term survival outcomes of advanced gastric cancer patients who achieved a pathological complete response with neoadjuvant chemotherapy: a systematic review of the literature Annals of Surgical Oncology 2015 22 3 787 792 10.1245/s10434-014-4084-9 25223927

[12] Mokdad AA Yopp AC Polanco PM Mansour JC Reznik SI Adjuvant Chemotherapy vs Postoperative Observation Following Preoperative Chemoradiotherapy and Resection in Gastroesophageal Cancer: A Propensity Score-Matched Analysis JAMA Oncology 2018 4 1 31 38 10.1001/jamaoncol.2017.2805 28975352 PMC5833647

[13] Becker K Mueller JD Schulmacher C Ott K Fink U Histomorphology and grading of regression in gastric carcinoma treated with neoadjuvant chemotherapy Cancer 2003 98 7 1521 1530 10.1002/cncr.11660 14508841

[14] Fonseca T Coimbra M Barbosa E Barbosa J Gastric cancer: histological response of tumor and metastatic lymph nodes for perioperative chemotherapy Cirugia y Cirujanos 2022 90 S2 36 41 10.24875/CIRU.21000657 36480751

[15] Schmid P Cortes J Dent R McArthur H Pusztai L Overall Survival with Pembrolizumab in Early-Stage Triple-Negative Breast Cancer New England Journal of Medicine 2024 391 21 1981 1991 10.1056/NEJMoa2409932 39282906

[16] Wang K Ma J Luo W Yin Q Zhang X Adjuvant osimertinib therapy guided by ctDNA-assessed MRD in resected EGFR-mutated stage IA-IIA non-small-cell lung cancer: a randomized clinical trial study protocol American Journal of Cancer Research 2024 14 11 5427 5433 10.62347/IFRH7248 39659920 PMC11626269

[17] Zhou Q Gampenrieder SP Frantal S Rinnerthaler G Singer CF Persistence of ctDNA in Patients with Breast Cancer During Neoadjuvant Treatment Is a Significant Predictor of Poor Tumor Response Clinical Cancer Research 2022 28 4 697 707 10.1158/1078-0432.CCR-21-3231 34862246 PMC9377752

[18] Liu DS Hall K Wong D Lee MMW Duong C 1402MO An international study evaluating pathological response to guide adjuvant FLOT chemotherapy in gastroesophageal cancer Annals of Oncology 2024 35 S879 10.1016/j.annonc.2024.08.1468

[19] Lin J-X Tang Y-H Lin G-J Ma Y-B Desiderio J Association of Adjuvant Chemotherapy With Overall Survival Among Patients With Locally Advanced Gastric Cancer After Neoadjuvant Chemotherapy JAMA Network Open 2022 5 4 e225557-e 10.1001/jamanetworkopen.2022.5557 35363268 PMC8976237

[20] Ikoma N Estrella JS Blum Murphy M Das P Minsky BD Tumor Regression Grade in Gastric Cancer After Preoperative Therapy Journal of Gastrointestinal Surgery 2021 25 6 1380 1387 10.1007/s11605-020-04688-2 32542556 PMC11957322

[21] Neves Filho EH de Sant’Ana RO Nunes LV Pires AP da Cunha MD Histopathological regression of gastric adenocarcinoma after neoadjuvant therapy: a critical review APMIS 2017 125 2 79 84 10.1111/apm.12642 28044374

[22] Bang YJ Kim YW Yang HK Chung HC Park YK Adjuvant capecitabine and oxaliplatin for gastric cancer after D2 gastrectomy (CLASSIC): a phase 3 open-label, randomised controlled trial Lancet 2012 379 9813 315 321 10.1016/S0140-6736(11)61873-4 22226517

[23] Wang X Zhao L Liu H Zhong D Liu W A phase II study of a modified FOLFOX6 regimen as neoadjuvant chemotherapy for locally advanced gastric cancer British Journal of Cancer 2016 114 12 1326 1333 10.1038/bjc.2016.126 27172250 PMC4984457

[24] Li ZY Koh CE Bu ZD Wu AW Zhang LH Neoadjuvant chemotherapy with FOLFOX: improved outcomes in Chinese patients with locally advanced gastric cancer Journal of Surgical Oncology 2012 105 8 793 799 10.1002/jso.23009 22189752

[25] Chen W Shen J Pan T Hu W Jiang Z FOLFOX versus EOX as a neoadjuvant chemotherapy regimen for patients with advanced gastric cancer Experimental and Therapeutic Medicine 2014 7 2 461 467 10.3892/etm.2013.1449 24396426 PMC3881068

